# FINANCIAL HARDSHIP IS ASSOCIATED WITH REPEATED STRESSORS AS MEASURED BY STRESSOR DIVERSITY

**DOI:** 10.1093/geroni/igae098.0346

**Published:** 2024-12-31

**Authors:** Agus Surachman

**Affiliations:** Drexel University, Philadelphia, Pennsylvania, United States

## Abstract

Financial hardship supplements traditional measures of socioeconomic status (i.e., education, income) to elucidate how the insufficiency of socioeconomic resources is experienced. Financial hardship may impact health by differentiating exposure to stressors in daily life. This study examined how three domains of financial hardship (i.e., material, behavioral, and psychological) relate to daily stressor concentration (e.g., experiencing many stressors that are all arguments versus fewer stressors spread across arguments, work stress, home overloads). Higher stressor concentration has been associated with poorer health and well-being across adulthood. The present analyses used data from the Midlife in the United States study wave 3 (2016-2020), in which 1,024 participants (ages = 43-90, 57% female) completed 8-day daily diary interviews. Measures of financial hardship include material indicators of poverty, healthcare, and financial aid; psychological perceptions of financial situation; and behaviors including payments, earnings, and spending. Daily stressor concentration was calculated using 1- the Gini diversity index. In a regression model accounting for age, sex, daily stressor exposure, and education, a higher total score of financial hardship was associated with higher stressor concentration (b = 0.07, SE = 0.03, p <.01). This result replicated across material (b = 0.26, SE = 0.09, p <.01) and psychological domains (b = 0.11, SE = 0.04, p <.01) of financial hardship, and the findings were exacerbated with older age (ps <.05). Experiencing repeated stressors in specific domains may be a mechanism by which insufficient economic resources can worsen health outcomes for adults as they age.

